# Dexamethasone drives macrophage repolarization linked to increased triple-negative breast cancer aggressiveness

**DOI:** 10.1038/s41419-025-08363-9

**Published:** 2025-12-19

**Authors:** Mohamed M. Shamekh, Birgitta Lindqvist, Ivan Nalvarte

**Affiliations:** 1 Department of Neurobiology, Care Sciences and Society, Division of Neurogeriatrics, Karolinska Institutet, Solna, Sweden; 2 Department of Biosciences and Nutrition, Karolinska Institutet, Huddinge, Sweden; 3 Department of Biochemistry, Faculty of Veterinary Medicine, Assiut University, Assiut, Egypt; 4 Department of Oncology-Pathology, Karolinska Institutet, Solna, Sweden

**Keywords:** Breast cancer, Cancer microenvironment, Cancer therapy

## Abstract

Glucocorticoids (GCs) are known for their anti-inflammatory potential, which includes the macrophage polarization into an anti-inflammatory and tissue remodeling state. GCs are routinely co-administered to cancer patients to alleviate the side effects of chemotherapy. However, it is not well known if GCs can modulate tumor-associated macrophages (TAMs) to promote tumor progression. Here, we show that dexamethasone (DEX) induces dose-dependent differentiation of THP-1 monocyte-derived anti-tumorigenic (M1) macrophages into pro-tumorigenic (M2-like) macrophages, even in the presence of M1 cues, and that DEX can repolarize fully differentiated M1 macrophages into an M2-like state. These macrophages have a cytokine profile similar to the pro-tumorigenic (M2) macrophages and can stimulate the proliferation and invasion of triple-negative breast cancer (TNBC) cells in vitro. DEX treatment of an orthotopic mouse model of TNBC attenuated paclitaxel-mediated tumor growth inhibition, increased M2-like TAMs in primary tumors, and enhanced lung metastasis. Transcriptomic analysis of DEX-treated M1 macrophages revealed not only transcriptomic overlap with M2 macrophages, but also with human breast cancer TAM transcriptomic data, and further to a specific TAM signature associated with aggressive estrogen receptor-negative breast cancer. Our study illustrates a remarkable macrophage repolarization plasticity upon DEX exposure that can promote tumorigenesis, warranting care in prescribing high doses of GCs to breast cancer patients, especially to those considered for chemotherapy.

## Introduction

Dexamethasone is a synthetic glucocorticoid steroid that is routinely administered to advanced cancer patients, including breast cancer patients undergoing chemotherapy, to manage side effects such as nausea, skin rashes, and inflammation [1, 2]. However, the effects of GCs on non-hematological tumor growth and metastasis are not well known. Studies have shown that GCs can both suppress and promote tumor growth, which appears to depend on cancer cell type and the timing and concentration of GC treatment. For instance, pre-operative GC administration can improve survival in pancreatic adenocarcinoma patients [3], and GC treatment inhibited growth and angiogenesis in a residual Lewis lung carcinoma model [4], as well as inhibited tumor cell migration and invasion in non-small cell lung cancer (NSCLC) upstream of VEGF signaling [5]. On the other hand, it has been demonstrated that GCs have pro-metastatic effects in the orthotopic 4T1 TNBC mouse model [6] as well as in the MDA-MB-231 breast cancer xenograft model [7]. Another study showed that DEX affects tumor growth and metastasis but in a dose-dependent manner, illustrating GCs as a double-edged sword where lower concentrations may inhibit breast cancer growth and metastasis, while higher doses promote breast cancer progression [8].

TAMs play an important role in the metastatic process of solid tumors. They are highly sensitive to GCs, which induce an anti-inflammatory (M2-like) macrophage phenotype that leads to a decrease in the expression of co-stimulatory molecules on macrophages, subsequently impairing T-cell activation and reducing the secretion of pro-inflammatory cytokines like IL-6 and TNF-α [9]. This shift towards an anti-inflammatory M2-like state can contribute to immune evasion of tumors, as TAMs become less effective in presenting antigens and activating T-cells [10] but stimulate tumor microenvironment remodeling and angiogenesis [11]. In this respect, it is imperative to ask whether GC treatment given with chemotherapy could polarize macrophages towards a pro-tumorigenic phenotype.

In this study, we show that DEX can polarize macrophages to an M2-like phenotype and that even fully M1-polarized macrophages can be repolarized by DEX to M2-like macrophages. We also show that DEX treatment of M1 macrophages promotes TNBC invasion capacity and proliferation, and that DEX, in combination with paclitaxel chemotherapy, increases the metastatic capacity in a 4T1 orthotopic TNBC mouse model. Finally, we show a surprisingly high overlap of DEX-repolarized macrophages with a human breast cancer TAM transcriptomic signature and identify key factors involved in the DEX-mediated M1 to M2 polarization. Our study warrants careful consideration of DEX treatment for breast cancer patients receiving paclitaxel.

## Results

### Glucocorticoids are needed for macrophage polarization

**Fig. 1:**
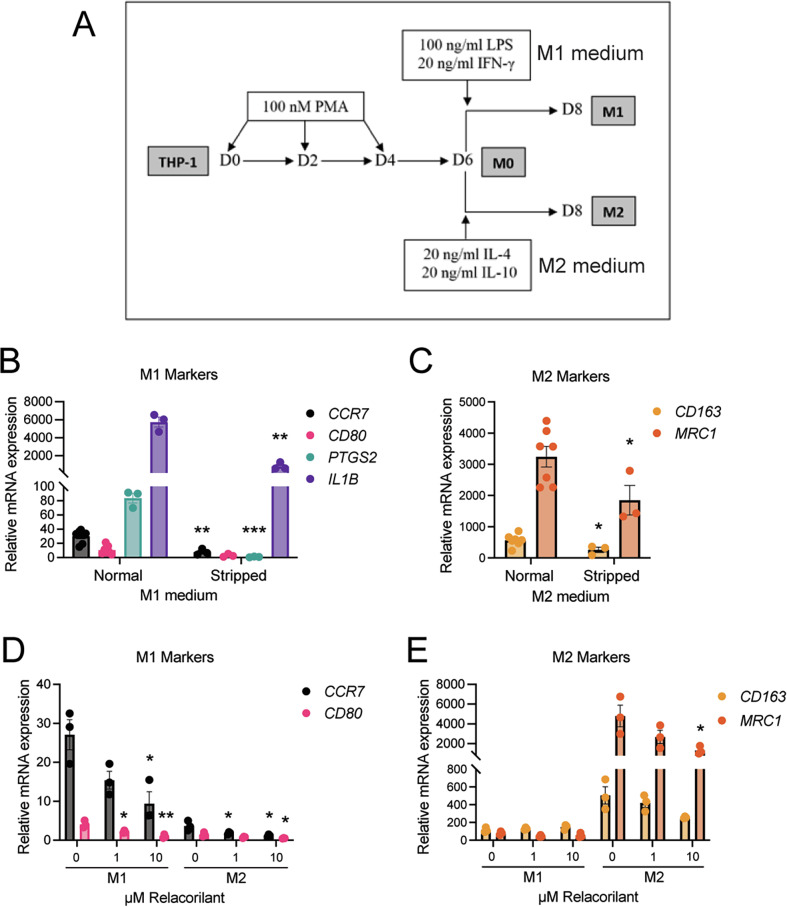
Glucocorticoids are needed for THP-1 monocyte differentiation. **A** Schematic of the differentiation protocol of THP-1 cells using 6 days of phorbol 12-myristate 13-acetate (PMA) to reach naïve macrophages (M), followed by 2 days of either lipopolysaccharides (LPS) and IFNγ treatment to reach M1 polarized macrophages, or IL-4 and IL-10 treatment to reach M2 polarized macrophages. **B** mRNA expression of the M1 markers *CCR7*, *CD80*, *PTGS2*, and *IL1B* (*n* = 3 [stripped], 7 [normal]) and **C** M2 markers *CD163* and *MRC1* (CD206) (*n* = 3 [stripped], 7 [normal]) upon culturing in normal or steroid-stripped M1 or M2 medium during differentiation. **D** Effect of increasing concentration of the selective GR antagonist relacorilant on mRNA expression of M1 markers *CCR7* and *CD80* (*n* = 3) and **E** M2 markers *CD163* and *MRC1* in M1 or M2 polarized macrophages. All mRNA expression was relative to untreated THP-1 cells and normalized to the housekeeping gene *HPRT1*. Comparisons were to the normal medium (**B**, **C**) or to the untreated (0 µM, **D**, **E**) condition. Bars represent mean ± SEM, *P < 0.05, **P < 0.01, ***P < 0.001. Statistical significance was determined using unpaired two-way ANOVA followed by Šídák’s (in **B** and **C**) multiple comparisons correction, or one-way ANOVA followed by Dunnett’s (in **D** and **E**) multiple comparisons correction.

**Fig. 2:**
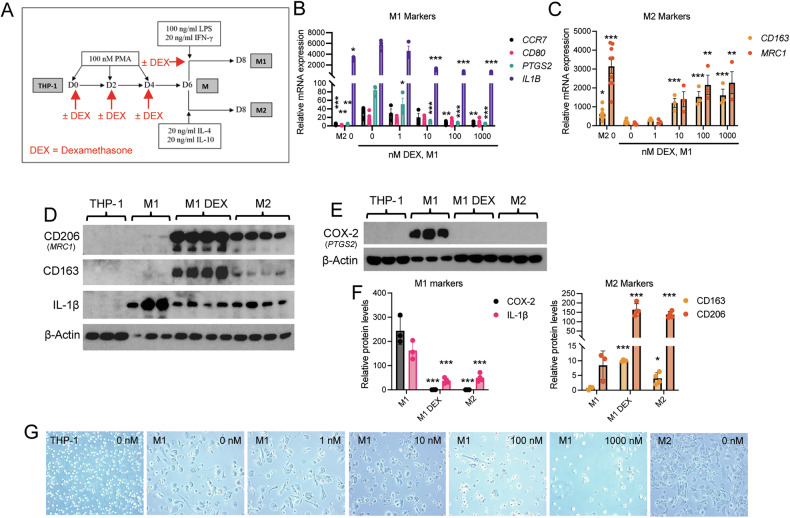
Dexamethasone suppresses M1 and stimulates M2 macrophage differentiation. **A** Differentiation protocol of THP-1 cells to non-polarized (M) and M1 or M2 polarized macrophages. DEX treatment was continuous during the M1 differentiation protocol. **B** mRNA expression of the M1 markers *CCR7*, *CD80*, *PTGS2* and *IL1B* (*n* = 3, 7 [M2 0, M1 0]), and **C** M2 markers *CD163* and *MRC1* (CD206) (*n* = 3, 7 [M2 0, M1 0]) upon M1 differentiation in the presence of increasing concentrations of DEX, or M2 differentiation in the absence of DEX. All mRNA expression was relative to untreated THP-1 cells and normalized to the housekeeping gene *HPRT1*, and comparisons were to M1 polarized macrophages at 0 nM DEX. **D** Western blot of the M2 markers CD206 and CD163, and the M1 markers IL-1β and **E** COX-2 in THP-1 cells, M1 and M2 polarized macrophages treated with vehicle (EtOH) or 100 µM DEX. **F** Western blot analysis of M1 (left) and M2 (right) markers relative to THP-1 cells (*n* = 3 and 4). Comparisons were to M1. **G** Cell morphological differences between THP-1 monocytes and M2 and M1 polarized macrophages upon indicated concentrations of DEX. Bars represent mean ± SEM in **B** and **C**, or ±SD in (**F**). *P < 0.05, **P < 0.01, ***P < 0.001. Statistical significance was determined using one-way ANOVA followed by Dunnett’s multiple comparisons correction.

GCs are present in the cell culture medium through the addition of FBS. To study the effect of low steroid levels on THP-1 monocyte differentiation and polarization into M1 and M2 macrophages, the cells were either cultured in normal FBS-containing growth medium or in steroid-deprived medium (with stripped FBS) prior and during the polarization (Fig. 1A). Both typical M1 macrophage markers (*CCR7*, *PTGS2*, and *IL1B*) and M2 macrophage markers (*CD163* and *MRC1*/CD206) were significantly downregulated upon steroid deprivation (Fig. 1B, C). To study the contribution of GCs, we used a highly selective GR antagonist, relacorilant (REL) [12], which resulted in a dose-dependent downregulation of both M1 and M2 markers (although not significant for *CD163*) (Fig. 2D, E). Additionally, the pan-macrophage marker *CD14* expression was downregulated upon steroid deprivation but not REL treatment, while *NR3C1*/GR expression was unchanged (Supplementary Fig. S2A, B). These data suggest that physiological GC levels are needed for macrophage polarization, and therefore, all subsequent experiments were performed in normal FBS-containing growth media.

### Dexamethasone suppresses M1 and stimulates M2 macrophage polarization

We next studied the effect of adding the GC analog DEX during the process of THP-1 monocyte differentiation to M1 macrophages (Fig. 2A). DEX treatment down-regulated mRNA expression of the typical M1 markers (Fig. 2B) and upregulated the M2 markers *CD163* and *MRC1*/CD206 compared to vehicle (EtOH)-treated M1 macrophages (Fig. 2C). Similarly, we saw a striking decrease of the protein levels of M1 specific markers IL-1β and COX-2 (cyclooxygenase-2, *PTGS2* gene product), while the typical M2 markers *MRC1*/CD206 and *CD163* were upregulated (Fig. 2D–F). Although *NR3C1*/GR mRNA expression was not changed, GR protein levels were increased in M2 macrophages and in the DEX-treated M1 macrophages compared to untreated M1 macrophages (Supplementary Fig. S2C–E). Furthermore, a more rounded and less granular phenotype of M1 differentiated macrophages was observed upon DEX treatment (Fig. 2G). These observations, combined, suggest that DEX suppresses M1 phenotype but promotes M2 phenotype, even in the presence of lipopolysaccharide (LPS) and interferon-γ (IFN-γ).

**Fig. 3:**
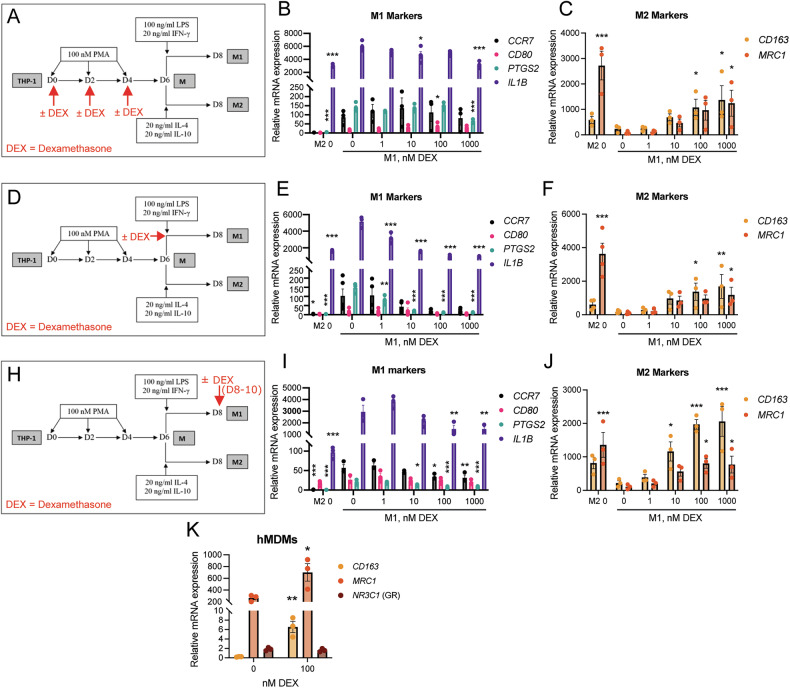
DEX can repolarize fully M1 polarized macrophages. **A** Schematic of DEX treatment during macrophage differentiation (day 0–6). **B** mRNA expression of the M1 markers *CCR7*, *CD80*, *PTGS2*, and *IL1B* (*n* = 3 [*CCR7*], 4), and **C**) M2 markers *CD163* and *MRC1* (*n* = 3). **D** Schematic of DEX treatment during macrophage M1 polarization (day 6–8). **E** mRNA expression of M1 markers (*n* = 3 [*PTGS2, IL1B*], 5 [*CCR7, CD80*]), and **F**) M2 markers (*n* = 3, 4 [M2 0, M1 0]). **H** Schematic of DEX treatment of M1 polarized macrophages (days 8–10). **I** mRNA expression of M1 markers (*n* = 3) and **J** M2 markers (*n* = 3). M2 macrophages were not DEX-treated, and comparisons were to M1 polarized macrophages at 0 nM DEX in (**A**)–(**J**). **K** mRNA expression of the M2 markers *CD163* and *MRC1*, and *NR3C1* (GR) in primary human peripheral blood monocyte-derived macrophages (hMDMs) after 2 days of vehicle or 100 nM DEX treatment. All mRNA expression was relative to untreated THP-1 cells and normalized to the housekeeping gene *HPRT1* in **B**, **C**, **E**, **F**, **I**, **J**, and relative to untreated CD14^+^ hPBM cells and normalized to *HPRT1* in K. Bars represent mean ± SEM. *P < 0.05, **P < 0.01, ***P < 0.001. Statistical significance was determined using one-way ANOVA followed by Dunnett’s multiple comparisons correction in **B**, **C**, **E**, **F**, **I**, **J**, and using Student’s unpaired *t*-test in (**K**).

To study if DEX treatment during a specific differentiation timepoint skews M1 macrophages towards expressing M2 markers, we first treated differentiating THP-1 cells with DEX for 6 days (Fig. 3A). This resulted in no major differences in M1 marker expression (Fig. 3B) but did increase M2 marker expression (Fig. 3C). However, treatment of differentiated macrophages (M) with DEX for 2 days (Fig. 3D) resulted in a dose-dependent decrease in M1 markers (Fig. 3E) and an increase in M2 marker expression (Fig. 3F). This suggests that exposure of naïve M macrophages to DEX polarizes macrophages to an M2-like state, even in the presence of M1-promoting factors (LPS and IFNγ). Next, we explored if DEX exposure can repolarize fully M1 polarized macrophages to express M2 markers (Fig. 3H). Indeed, DEX exposure, especially at ≥100 nM, significantly decreased the M1 markers *PTGS2* and *IL1B* (Fig. 3I) and upregulated the M2 markers *CD163* and *MRC1*/CD206 (Fig. 3J), suggesting that even fully polarized M1 macrophages have a plasticity to change polarization upon DEX exposure. To validate these results, we exposed M1 primary human monocyte-derived macrophages (hMDMs) to 100 nM DEX for 2 days, which confirmed that DEX upregulates the M2 markers *CD163* and *MRC1*/CD206 (Fig. 3K), even in these non-leukemic origin macrophages. We could not observe any difference in *NR3C1*/GR or *CD14* mRNA expression upon DEX treatments in M1 macrophages (Supplementary Fig. S2F–H).

### Dexamethasone-treated M1 macrophages have a secretory profile similar to M2 macrophages

**Fig. 4:**
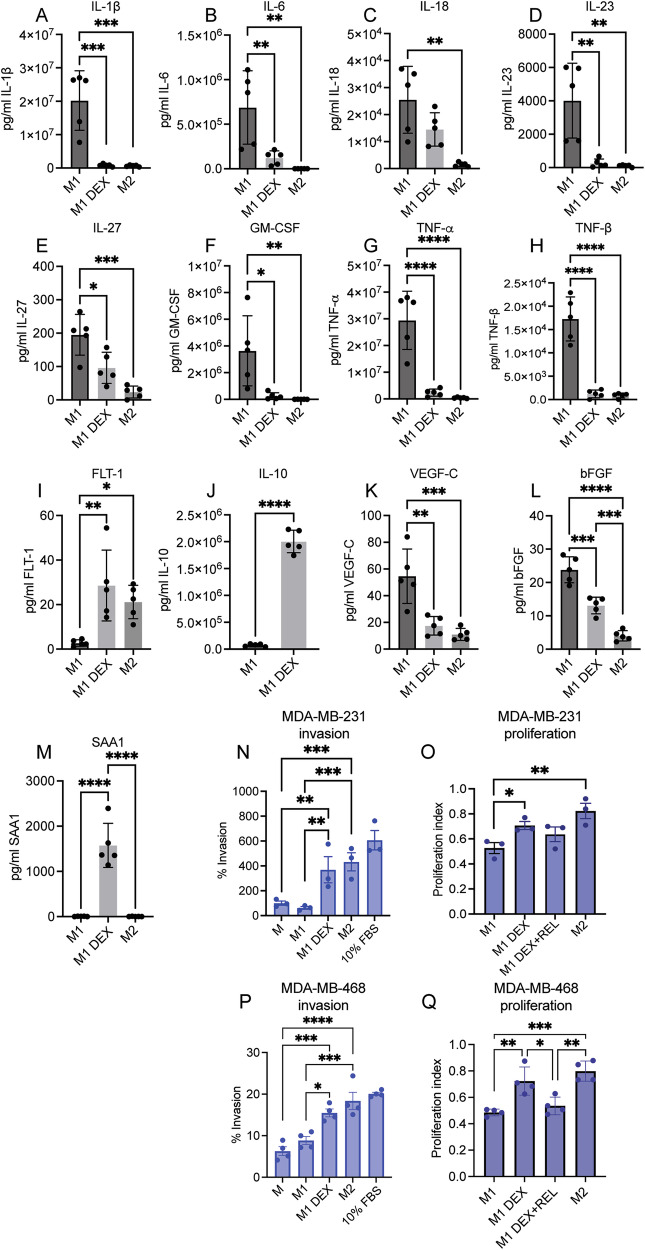
Secretory profile of DEX-treated M1 macrophages. Immunoassay analysis of **A** IL-1β, **B** IL-6, **C** IL-18, **D** IL-23, **E** IL-27, **F** GM-CSF, **G** TNF-α, **H** TNF-β, **I** FLT-1, **J** IL-10, **K** VEGF-C, **L** bFGF, and **M** SAA1, upon 8 days of 100 nM DEX (M1 DEX) or vehicle (EtOH) treatment during TPH-differentiation to M1 polarized macrophages, or vehicle (EtOH) treatment during differentiation to M2 polarized macrophages (*n* = 5). **N** MDA-MB-231 cell Matrigel invasion in response to conditioned medium from unpolarized macrophages (M), M1 polarized macrophages (M1), DEX-treated polarized macrophages (M1 DEX), M2 polarized macrophages (M2), and to 10% FBS-containing medium (positive control) (*n* = 3). **O** Effect on MDA-MB-231 proliferation upon 2-day incubation with conditioned medium from M1, M1 DEX, M1 DEX medium with 10 µM GR-inhibitor relacorilant (REL), and medium from M2 macrophages (n = 3). **P** Matrigel invasion and **Q** proliferation of MDA-MB-468 cells in response to the same conditioned medium as in **N** and **O** (*n* = 4). Bars represent mean ± SD in **A**–**M**, or ±SEM in (**N**, **O**, **P**, **Q**). *P < 0.05, **P < 0.01, ***P < 0.001, ****P < 0.0001. Statistical significance was determined using one-way ANOVA followed by Tukey’s multiple comparisons correction, or an unpaired Student’s *t*-test in (**J**).

Depending on their polarization, macrophages secrete pro-inflammatory and tumor-suppressing (M1) or anti-inflammatory and tissue remodeling (M2) factors. The latter are associated with M2 TAMs, evading immune cell targeting and promoting tumor cell metastasis. We could observe that DEX treatment for 8 days during TPH-1 differentiation to M1 macrophages as depicted in Fig. 2A (M1 DEX), resulted in decreased secretion of the typical M1 factors IL-1β, IL-6, (IL-18 not significant), IL-23, IL-27, GM-CSF, TNF-α, and TNF-β (Fig. 4A–H) into the media compared to the vehicle (EtOH)-treated M1 macrophages, and had more similar cytokine profile to vehicle-treated M2 macrophages. Oppositely, secretion of the M2 factors FLT-1 and IL-10 into the media was increased in M1 DEX condition compared to the vehicle-treated M1 condition (Fig. 4I, J), while VEGF-C and bFGF levels were decreased in the M1 DEX condition (Fig. 4K, L). We could also observe that serum amyloid A1 protein (SAA1) was only secreted in the M1 DEX condition (Fig. 4M), similarly to findings in a previous report [13]. No effect of DEX was observed on macrophage apoptosis (Supplementary Fig. S3A). However, DEX slightly increased both monocyte apoptosis (Supplementary Fig. S3B) and proliferation (Supplementary Fig. S3C).

To test how a TNBC cell line responds to these secretory profiles, we exposed mesenchymal-like TNBC MDA-MB-231 cells to 2-day-old growth medium from DEX-treated (M1 DEX) or vehicle-treated M1 polarized macrophages and vehicle-treated M2 polarized macrophages. We observed a drastic increase in MDA-MB-231 cell Matrigel invasion towards M1 DEX medium compared to medium from vehicle-treated M1 macrophages, similar to that of medium from vehicle-treated M2 macrophages (Fig. 4N). Furthermore, M1 DEX medium also increased MDA-MB-231 cell proliferation compared to medium from vehicle-treated M1 cells, which was slightly attenuated by the specific GR antagonist relacorilant (Fig. 4O). Similar effects were observed with the more basal-like breast cancer cell line MDA-MB-468 (Fig. 4P, Q) (although it had an overall less invasive phenotype), suggesting that the DEX–macrophage–TNBC interaction may be a general phenomenon in TNBC cells. Combined, these data suggest that DEX promotes an M2-like phenotype in M1 macrophages, which can promote breast cancer cell invasion and proliferation.

### Dexamethasone increases tumor size and lung metastasis in the 4T1 orthotopic mouse model of triple-negative breast cancer

**Fig. 5:**
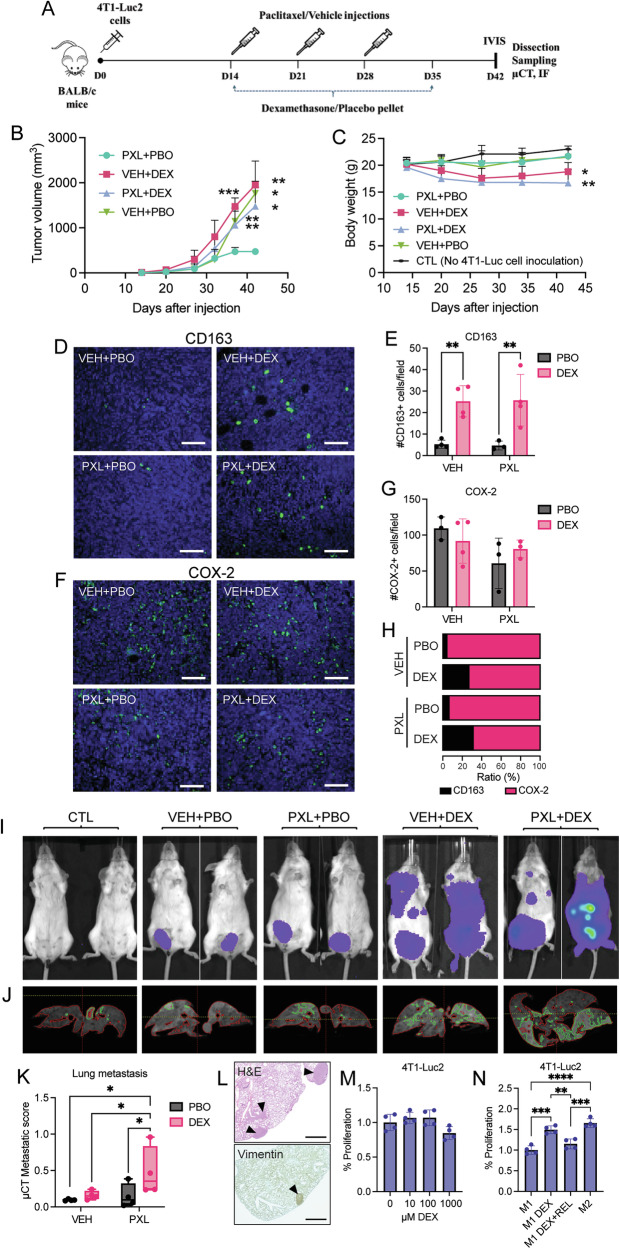
DEX elevates the M2 to M1 macrophage ratio in primary breast tumors and promotes lung metastasis in paclitaxel-treated mice. **A** Schematic of 4T1-Luc2 cell injection and treatment regimen of BALB/c female mice. **B** Tumor volume (mm^3^) at indicated days post 4T1-Luc2 tumor cell injection. Significant differences were observed between the paclitaxel (PXL) group and dexamethasone (DEX)-treated groups, as well as the placebo (PBO) group at day 37 (*n* = 4, 5 [PXL + PBO]) and 42 (*n* = 3, 4 [PXL + PBO]) post-injection. Vehicle (VEH) was injected in all experimental groups not treated with paclitaxel. **C** Body weight (g) of test groups at days 15–42 post-injection. Control group (CTL) was not subjected to cell injection or any treatment. **D** Representative images from immunohistochemical analysis of CD163+ macrophage infiltration into primary mammary tumors in mice treated as indicated (scale bar = 100 µm). **E** Quantification of CD163+ cells (in **D**) (*n* = 3 [PXL + PBO], 4). **F** Representative images from immunohistochemical analysis of COX-2+ macrophage infiltration into primary mammary tumors in mice treated as indicated (scale bar = 100 µm). **G** Quantification of COX-2 cells (in **F**) (*n* = 3, 4 [VEH + DEX]). **H** Ratio (%) CD163+ and COX-2+ cells in primary tumors. **I** Qualitative assessment of primary tumors and metastases by IVIS imaging of 4T1-Luc2 cells after D-luciferin injection. **J** Representative images from µCT scans (red-circled area = normal lung parenchyma; green-circled area = tumor tissue and blood vessels). **K** Quantification of µCT metastatic score in the different treatment groups compared to CTL-mice (*n* = 4). **L** Representative images of H&E (left) and vimentin (right) stained lung slides from PXL + DEX-treated mice (scale bar = 1 mm). **M** Proliferation of 4T1-Luc cells in the presence of increasing amounts of DEX. Shown as percent relative to 0 µM DEX treatment (*n* = 4). **N** Proliferation of 4T1-Luc cells in response to conditioned medium from treated macrophages (as in Fig. 4N) (*n* = 4). Data represent the means ± SD in **B**, **C**, **E**, **G**, **M**, and **N**, and bars represent median ± min to max values in (**K**). *P < 0.05, **P < 0.01, ***P < 0.001, ****P < 0.0001. Statistical significance was determined using one-way ANOVA followed by Dunnett’s correction for multiple comparisons in **B**–**D**, **M**, and **N**, and by two-way ANOVA followed by uncorrected Fisher’s LSD test for multiple comparisons in (**E**), (**G**), and (**K**).

To study the effect of DEX treatment on TNBC growth and metastasis, we injected 4T1-Luc2 triple-negative mouse mammary cancer cells into the 4th mammary fat pad of BALB/c mice. Tumors were palpable on day 14 when mice were treated with or without paclitaxel (PXL, 20 mg/kg or vehicle) and with or without DEX (DEX s.c. pellets, 0.125 mg/day release, or placebo pellets) (Fig. 5A). While PXL effectively inhibited tumor growth, the combined PXL + DEX treatment abolished this inhibition (Fig. 5B). There was no significant difference between the DEX (VEH + DEX) group and the placebo (VEH + PBO) group at any of the indicated time points (Fig. 5B). Largest body weight decrease was seen in PXL + DEX and DEX alone (VEH + DEX)-treated mice (Fig. 5C). When analyzing the polarization of infiltrating macrophages in primary tumors, we could observe that DEX treatment significantly increased the number of CD163-positive macrophages (Fig. 5D, E), while it had no statistically significant effect on COX-2 positive macrophages (Fig. 5F, G). We also observed that the ratio of CD163 to COX-2-positive macrophages was increased in the primary tumors upon DEX treatment (Fig. 5H). We next analyzed metastases qualitatively using an in vivo imaging system (IVIS) (Fig. 5I) and lung metastatic lesions quantitatively with high-resolution µ-computer tomography (µCT) (Fig. 5J, K) on day 42 after cell injections. The PXL + DEX group showed strikingly higher lung metastatic burden compared to any other treatment (Fig. 5K, L). Next, we assessed 4T1-Luc2 cell proliferation in vitro in response to DEX. Although no direct effect of DEX on 4T1-Luc2 cell proliferation could be observed (Fig. 5M), conditioned medium from DEX-treated M1 macrophages promoted 4T1-Luc2 proliferation, which could be mitigated in conditioned medium from M1 macrophages co-treated with DEX and the GR inhibitor relacorilant (Fig. 5N). This suggests that the DEX effect on 4T1-Luc2 cell tumorigenic effects at distal sites in vivo could be mediated via interaction with macrophages, which may mitigate paclitaxel growth inhibition.

### Transcriptomic analysis of dexamethasone-treated M1 macrophages and comparison to tumor-associated macrophages from human breast cancer

**Fig. 6:**
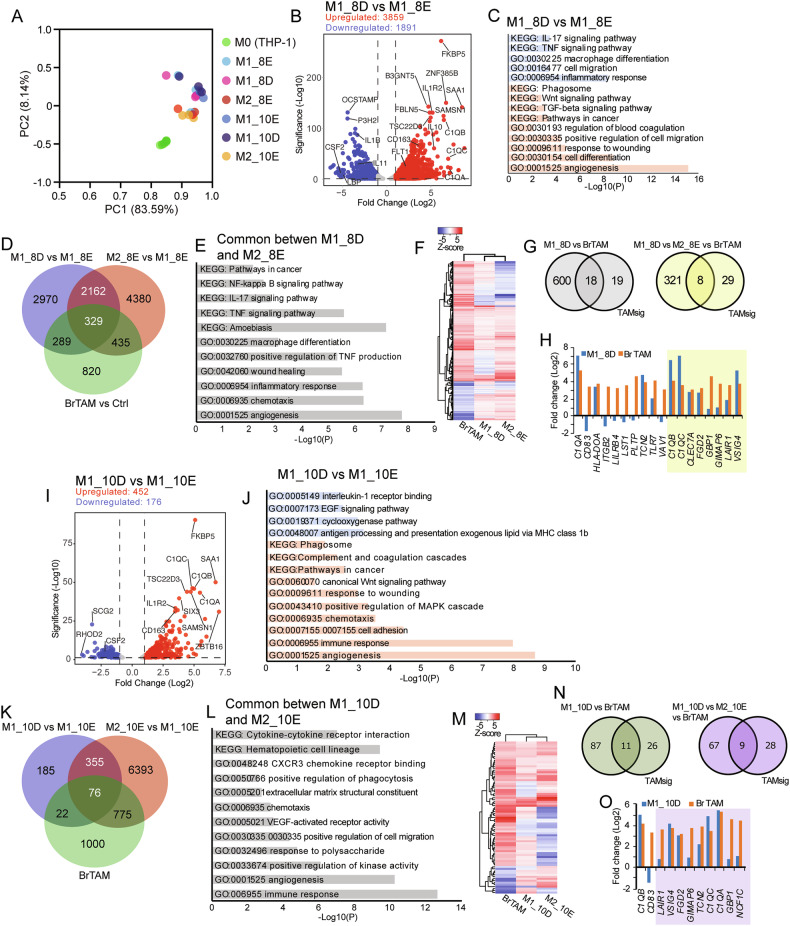
Transcriptomic profiling of DEX-treated macrophages and comparison to human breast tumor-associated macrophages. **A** Principal component (PC) analysis of normalized counts from THP-1 monocytes (M0), vehicle-treated M1 polarized macrophages (M1_8E), DEX-treated M1 polarized macrophages (M1_8D), vehicle-treated M2-polarized macrophages (M2_8E), 2-day vehicle-treated fully M1 polarized macrophages (M1_10E), 2-day DEX-treated fully M1 polarized macrophages (M1_10D), and 2-day vehicle-treated fully M2 polarized macrophages (M2_10E). **B** Volcano plot of the differentially expressed genes (DEGs) between M1_8D and M1_8E macrophages (cutoff Log2FC > 1, adjusted *P*-value < 0.05, FDR < 0.05). **C** Functional annotation of the DEGs between M1_8D and M1_8E macrophages (blue: downregulated, red: upregulated). **D** Venn diagram of the commonly regulated transcripts between M1_8D and M2_8E macrophages, and the DEGs from breast cancer tumor-associated macrophages (BrTAM). **E** Functional annotation of 2491 commonly regulated DEGs between M1_8D and M2_8E macrophages. **F** Hierarchical clustering of the DEGs between BrTAM, M1_8D, and M1-8E macrophages. Expression values are *Z*-score-transformed and clustered using complete linkage and Euclidean distance. **G** Venn diagram of commonly regulated transcripts between M1_8D, BrTAM, and a 37-gene TAM signature (TAMsig) (left), and between M1_8D, M2_8E, BrTAM, and TAMsig (right). **H** Bar plot of the common DEGs between M1_8D, BrTAM, and TAMsig, of which 8 DEGs were also common to M2_8E (highlighted in yellow) (FDR < 0.05). **I** Volcano plot of the DEGs between M1_10D and M1_10E macrophages (cutoff Log2FC > 1, adjusted *P*-value < 0.05, FDR < 0.05). **J** Functional annotation of the DEGs between M1_10D and M1_10E macrophages (blue: downregulated, red: upregulated). **K** Venn diagram of the commonly regulated transcripts between M1_10D and M2_10E macrophages, and the DEGs from BrTAM. **L** Functional annotation of 431 commonly regulated DEGs between M1_10D and M2_10E macrophages. **M** Hierarchical clustering of the DEGs between BrTAM and M1_10D and M2_10E macrophages. Expression values are *Z*-score-transformed and clustered using complete linkage and Euclidean distance. **N** Venn diagram of the commonly regulated transcripts between M1_10D, BrTAM, and TAMsig (left), and between M1_10D, M2_10E, BrTAM, and TAMsig (right). **O** Bar plot of the common DEGs between M1_10D, BrTAM, and TAMsig, of which 9 DEGs were also common to M2_10E (highlighted in blue) (FDR < 0.05). Please refer to Supplementary Tables S3–S5 for details.

Given the drastic effects of DEX on macrophage M1 repolarization to an M2-like phenotype, we performed bulk RNA-sequencing to explore the transcriptomic effects that may underlie the phenotypic change. For this, we used M1 macrophages treated with 100 nM DEX for 8 days during differentiation (M1_8D) in addition to M1 and M2 macrophages treated with vehicle control during differentiation (ethanol, E: M1_8E and M2_8E) as depicted in Fig. 2A, or macrophages treated with DEX for 2 days only after full M1 differentiation (M1_10D) in addition to M1 and M2 macrophages treated with vehicle control (M1_10E and M2_10E) as depicted in Fig. 3H. Principal component analysis (PCA) and hierarchical clustering showed that M1 and M2 macrophages clustered separately, largely irrespective of DEX treatment, and separately from THP-1 monocytes (M0) (Fig. 6A, Supplementary Fig. S4). Analysis of differentially expressed genes (DEGs) between M1_8D and M1_8E revealed 3859 upregulated and 1891 downregulated genes (cutoff Log2FC > 1, adjusted *P*-value < 0.05, FDR < 0.05), including upregulation of genes associated with M2 phenotype (e.g. *C1QA*, *C1QB*, *C1QC*, *SAA1, IL10, FLT1*, and *CD163*) and downregulation of genes associated with M1 phenotype (e.g. *IL1B*, *IL11*, *LBP*, and *CSF2*) (Fig. 6B). Pathway analysis of differently regulated genes revealed downregulation of pro-inflammatory pathways such as IL-17 and TNF-signaling pathways, and upregulation of M2-associated pathways such as phagosome, TGF-beta signaling and angiogenesis (Fig. 6C). We could detect 2491 commonly regulated genes between M1_8D and M2_8E compared to M1_8E (Fig. 6D). Commonly regulated pathways between these included regulation of NF-kappa B, IL-17, and TNF signaling, as well as macrophage differentiation, wound healing, chemotaxis and angiogenesis (Fig. 6E), suggesting phenotypic commonalities between DEX-treated M1 macrophages and M2 macrophages. We next explored if the transcriptomic changes upon DEX treatment of M1 macrophages overlapped with published [14] human transcriptomic data from tumor-associated macrophages in breast cancer (BrTAM). 618 DEGs in M1_8D overlapped with BrTAM, of which 329 also overlapped with M2_8E macrophages (Fig. 6D). Hierarchical clustering showed some distinct overlapping patterns of M1_8D with both M2_8E and BrTAM DEGs (Fig. 6F). To explore if the M1_8D cells further linked with clinical prognosis, we used a published human 37-gene transcriptomic TAM signature (TAMsig) associated with high tumor grade (high colony stimulating factor 1, CSF1, response signature), decreased estrogen and progesterone receptor expression, and high mutation rate [14]. Of the 618 DEGs that overlapped between M1_8D and BrTAM, 18 overlapped with the 37 TAMsig genes (48.6%), and of the DEGs that overlapped between M1_8D, BrTAM, and M2_8E, 8 genes overlapped with TAMsig (21.6%) (Fig. 6G); thus, a strikingly high overlap. These 8 genes all showed similar expression directions as in the TAMsig (Fig. 6H).

Since our data suggest that even fully polarized M1 macrophages can repolarize into M2-like macrophages upon DEX treatment (Fig. 3H–J), we compared the transcriptome of fully polarized M1 macrophages treated de novo with DEX for 2 days to M2 macrophages (M1_10D vs. M2_10E). We could detect 452 upregulated and 176 downregulated genes (cutoff Log2FC > 1, adjusted *P*-value < 0.05, FDR < 0.05), including upregulation of genes associated with M2 phenotype (e.g. *C1QA*, *C1QB*, *C1QC*, *SAA1*, *CD163* and *TSC22D3*), as well as downregulation of M1-associated genes (e.g. *CSF2* and *RHOD2*) (Fig. 6I). However, we could also detect that genes associated with M1 macrophages such as *SAMSN1* and *ZBTB16* were upregulated upon DEX treatment suggesting a mixed M1-M2 state. Pathway analysis revealed downregulation of typical M1 signaling, including IL-1 receptor binding and COX-2 signaling, and upregulation of typical M2 signaling, such as tissue remodeling, including response to wounding and angiogenesis, as well as phagocytosis and cancer-associated pathways (Fig. 6J). We could detect 431 commonly regulated genes between M1_10D and M2_10E compared to M1_10E (Fig. 6K). Common pathways between these included regulation of tissue remodeling (e.g., angiogenesis, cell migration, phagocytosis, and extracellular matrix structure), chemotaxis, and immune response (Fig. 6L). 98 of the M1_10D DEGs overlapped with the BrTAM dataset, of which 76 also overlapped with the M2_10E signature (Fig. 6K). Hierarchical clustering of the 98 DEGs showed clustering patterns suggestive of an intermediate state of M1_10D cells between M2_8E cells and BrTAMs (Fig. 6M). Of the 98 DEGs overlapping with BrTAM, 11 overlapped with TAMsig (29.7% of TAMsig), and of the DEGs that overlapped between M1_10D, BrTAM, and M2_10E, 9 genes overlapped with TAMsig (24.3%) (Fig. 6N) and had similar expression direction as in the TAMsig (Fig. 6O). These transcriptomic data suggest that both DEX treatment during macrophage differentiation and polarization, as well as de novo DEX treatment of fully polarized M1 macrophages, change the transcriptomic profile of M1 macrophages towards a TAM profile associated with aggressive breast cancer.

### Targeting DEG candidates inhibits the DEX-mediated M1 to M2 shift

**Fig. 7:**
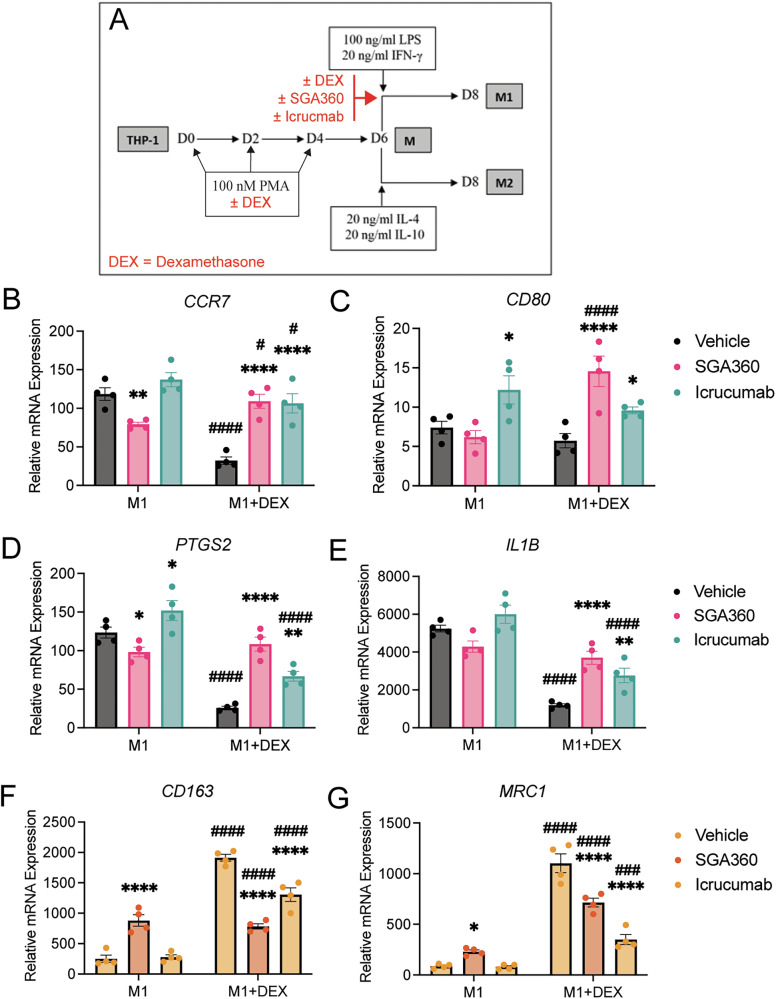
Targeting SAA1 and FLT-1 modulates DEX-induced M1 to M2 shift. **A** Schematic of DEX treatment during macrophage M1 polarization (days 6–8) in the presence of 10 µM SAA1 inhibitor SGA360 or 30 µg/ml FLT-1 neutralizing antibody Icrucumab. mRNA expression of the M1 markers **B** *CCR7*, **C** *CD80*, **D** *PTGS2*, and **E** IL1B, as well as the M2 markers **F** *CD163*, and *MRC1* (*n* = 4). Bars represent mean ± SEM. *P < 0.05, **P < 0.01, ***P < 0.001, ****P < 0.0001 relative to respective vehicle control, and #*P* < 0.05, ##*P* < 0.01, ###*P* < 0.001, ####*P* < 0.0001 relative to the respective M1 condition. Statistical significance was determined using two-way ANOVA followed by Šidák’s multiple comparisons correction.

Two interesting genes significantly upregulated in the DEX-treated M1 macrophages were *SAA1* and *FLT1* (Figs. 4 and 6B, and Supplementary Table S4), which participate in inflammatory responses and tissue remodeling, respectively [15, 16]. To test if these gene products are involved in the DEX-mediated M1 to M2 shift, THP-1 cells were treated with 100 nM DEX during macrophage differentiation for 6 days followed by co-treatment with the SAA1 inhibitor SGA360 or FLT-1 neutralizing antibody Icrucumab for 2 days as depicted in Fig. 7A. We observed an increase in classical M1 markers *CCR7*, *CD80*, *PTGS2*, and *IL1B* upon combined SGA360 and DEX treatment during M1 polarization (Fig. 7B–E). Similar effects were not observed in the M1 condition, where SAA1 inhibition instead led to a slight decrease in M1 marker expression (Fig. 7B). Supporting these results, we observed that M2 markers *CD163* and *MRC1* were downregulated with combined DEX and SGA360 treatment (Fig. 7F, G). However, SGA360 treatment alone in M1 macrophages led to increased M2 marker expression (Fig. 7F, G). This suggests that the effect of SAA1 is context-specific and, in the absence of DEX, can be pro-inflammatory. Inhibition of FLT-1 (VEGFR-1) with Icrucumab during M1 polarization resulted in a slight increase in M1 markers in the M1 condition, but a much stronger increase in DEX-treated M1 macrophages (Fig. 7B–E). In addition, FLT-1 inhibition resulted in significant downregulation of the M2 markers *CD163* and *MRC1* only in DEX-treated M1 macrophages (Fig. 7F, G). These data suggest that the DEX-mediated M1 to M2 shift can, to a significant extent, be prevented by inhibiting key mediators of macrophage function and polarization.

## Discussion

GCs are steroid hormones with important physiological functions related to stress, metabolism, and anti-inflammatory effects. GCs are therefore routinely used in the treatment of lymphomas and leukemias, but they are also administered to cancer patients to alleviate chemotherapy symptoms, such as nausea, skin rashes, and inflammatory side effects [1, 2]. However, studies have shown that GCs at high dosages (as in those used to mitigate side effects of chemotherapy) may promote non-hematological tumor growth and metastasis [6–8]. These studies have shown that GCs can directly modulate tumor cell proliferation and invasion, thereby aggravating tumor progression. In breast cancer patients, it was shown that activation of GR was associated with poor prognosis and shorter relapse-free survival, but only in patients with estrogen receptor-negative (ER-) breast cancer, and that GR activation increased epithelial-to-mesenchymal transition (EMT) [17]. It was also shown that GR activation in a mouse xenograft model of TNBC drastically decreased paclitaxel chemotherapy response due to upregulation of the anti-apoptotic machinery in the tumor cells [18].

TAMs are well-known modulators of the tumor microenvironment. Depending on their polarization, they can excrete cues in their M1 state to promote cytotoxicity and inflammatory clearance of tumor cells, or in their M2 state, promote a supportive microenvironment for tumor cell growth and extravasation [19, 20]. Reciprocal signals between TAMs and the tumor cells stimulate M2 macrophage polarization and secretion of anti-inflammatory signals to facilitate tumor immune evasion [21–23].

However, macrophages are also well-known anti-inflammatory mediators of GCs [24], which in the context of TAMs may thus promote tumor growth and metastasis. Nevertheless, there are, to our knowledge, no studies on the direct effect of exogenous GCs on TAMs, which is surprising since GCs are commonly co-administered with chemotherapy. In our current study, we show that GCs are needed for proper THP-1 monocyte differentiation into macrophages as well as for their proper M1 or M2 polarization (Fig. 1). We also show that macrophage differentiation and polarization is highly plastic, as DEX treatment at clinically relevant concentrations [25, 26] promotes M1 macrophages into an M2-like phenotype in a dose-dependent manner even under M1 cues (Fig. 2), and even if these were already fully M1 polarized (Fig. 3). Similar effects of DEX on THP-1-derived macrophage re-polarization were observed in the context of fungal infections where DEX changed macrophage metabolic activity and dampened their pro-inflammatory response [27]. We observed that DEX-induced re-polarization also applies to human peripheral blood-derived M1 macrophages of non-leukemic origin (Fig. 3K), which, in phenotype and functionality, have been shown to be very similar to bone-marrow-derived macrophages [28], suggesting similar responses across macrophages derived from different anatomical niches. Furthermore, we observed that the cytokine profile of DEX-treated M1 macrophages was similar to M2 macrophages and promoted TNBC cell invasion and growth in vitro (Fig. 4). Interestingly, using an orthotopic mouse model of TNBC, we observed that DEX treatment (131.25 mg/kg over 3 paclitaxel treatment cycles, which in view of mice higher metabolic rate is comparable to human dosages) decreased paclitaxel chemotherapy response of tumor growth, similar to what was reported by Pang and colleagues [18], with elevated metastatic potential and increased M2-like macrophages in the primary tumors (Fig. 5). The latter was likely due to increased M2-like macrophage infiltration into the tumor rather than M1-to-M2 transition, since the number of M1 macrophages did not change in the tumors. In contrast, another study using overall higher DEX dosages (400 mg/kg over 3 weeks) compared to our study reported an increase in M1 markers and a decrease in M2 markers in a model of Lewis lung carcinoma without any additional chemotherapeutic agent [29]. Yet another study [30] found that rat blood monocytes treated in vitro with higher DEX doses than in our study (10–100 µM for 48 h, which is higher than clinical dosages) polarized monocytes towards an M2-like macrophage phenotype. Co-treatment with antigen could increase M2 marker expression [30], suggesting that lower DEX doses in combination with antigen stimulation would probably also be effective for M2 polarization. Although we had a different experimental setup, we observed that co-treatment of DEX and M1 cues of mature macrophages indeed had stronger effects (Fig. 3D–F) than the treatment of differentiating monocytes with DEX (Fig. 3A–C). Thus, differences in in vitro and in tumor models, as well as DEX timing and dosage, may account for different macrophage responses. When comparing the transcriptomic profile of DEX-stimulated M1 macrophages, we could observe activation of genes and pathways normally associated with the M2 phenotype (Fig. 6). Although PCA and hierarchical clustering analysis showed partial overlaps, when comparing the data to human breast cancer TAM transcriptomic data and further to a TAM-specific signature associated with aggressive ER- breast cancer, we could detect surprisingly high overlap to our DEX-treated M1 macrophages (Fig. 6D–H). DEX treatment of fully M1 polarized macrophages showed a similar, although less, overlap with the human breast cancer TAM signature (Fig. 6K–O).

From the ELISA and transcriptomic data, we chose two genes for functional follow-up, *SAA1* and *FLT1*, which were highly upregulated in DEX-treated M1 macrophages (Figs. 4, 6A, and Supplementary Table S4). SAA1 is a known GR target gene [31] which can mediate context-specific macrophage responses, both proinflammatory acute responses and anti-inflammatory reparative effects [13, 16, 32]. In our study, inhibition of SAA1 with the small molecule drug SGA360 resulted in a significant increase in M1 markers and a decrease in M2 markers in DEX-treated macrophages (Fig. 7), suggesting that SAA1 participates in the DEX-mediated M1 to M2 shift. Interestingly, SAA1 inhibition also slightly decreased M1 markers and increased M2 markers in the M1 condition in the absence of DEX, suggesting that SAA1 can regulate inflammatory responses differently in the absence or presence of DEX, likely in concert with other proinflammatory factors such as IL-6 and TNF-α [13, 31]. Inhibition of FLT-1, a soluble tissue-remodeling factor, with the neutralizing antibody Icrucumab increased the expression of M1 markers and decreased M2 markers, especially in the presence of DEX (Fig. 7). Combined, these data suggest that key factors such as SAA1 and FLT-1 participate in the DEX-mediated M1 to M2 shift.

Our study supports that DEX can promote breast cancer growth and metastasis, especially under paclitaxel treatment. This warrants careful assessment of GC use as co-administration with chemotherapy. Since essentially all breast cancer patients are co-administered GCs during chemotherapy, there are no case-control data available on the negative effects of GCs on breast cancer prognosis. However, as mentioned above, Pan et al. [17] demonstrated that high GR levels in ER− breast cancer were associated with poor prognosis. Although they could not see any interaction between chemotherapy, GR levels, and disease progression, it is likely that all chemotherapy patients in their study also received GCs, masking such interactions. Finally, timing, dosage, and duration of GC treatment may have different effects on breast cancer progression, where lower GC doses given to less progressed tumors may have antitumoral activities, while higher GC doses given to more advanced tumors may promote cancer progression. Our data should thus be followed up with studies addressing GC dosage and timing, as well as with studies using other cancer types and other chemotherapies. But until then, our study warrants care in prescribing high-dose GC treatments to breast cancer patients, especially those considered for paclitaxel treatment.

## Materials and methods

### Cell culture and macrophage differentiation

The human monocytic cell line THP-1, human triple-negative breast cancer (TNBC) MDA-MB-231 cells, human TNBC MDA-MB-468 cells, and the mouse TNBC luciferase-expressing 4T1-Luc2 cells were obtained from ATCC (#TIB-202, #HTB-26, #HTB-132, #CRL-2539-LUC2; respectively) and propagated in ATCC-modified RPMI-1640 medium supplemented with 10% fetal bovine serum (FBS) (both from Gibco; #A1049101, #10500064; respectively). THP-1 monocytes were grown in suspension and differentiated into M1- or M2-polarized macrophages according to the protocol shown in Fig. 1A. Induction to non-polarized macrophages (M) was done with phorbol 12-myrisate 13-acetate (PMA, 100 nM) for 6 days, followed by 2-day polarization to M1 or M2 macrophages with interferon-gamma (IFNγ, 20 ng/ml) and lipopolysaccharides from *E. Coli* (LPS, 100 ng/ml), or IL(interleukin)-4 (20 ng/ml) and IL-10 (20 ng/ml); respectively (all from Merck; #P8139, #IF002, #L2630, #I4269, #SRP3071; respectively). The cells were treated with indicated concentrations of dexamethasone (Merck; #D4902), the glucocorticoid receptor (GR)-specific antagonist relacorilant (CORT125134, kind gift from Corcept Therapeutics; Menlo Park, CA, USA), the SAA1 inhibitor SGA360 (10 mM, MedChemExpress; #HY-122208), and/or the FLT-1 neutralizing antibody Icrucumab (30 µg/ml, MedChemExpress; #HY-P99364, and control antibody #HY-P99003). In addition, primary CD14^+^ human peripheral blood monocytes (hPBMs) were purchased from PromoCell (#C-14110) as a single-donor cell pellet stored in RNA*later*^®^. Primary human monocyte-derived macrophages (hMDMs) were also purchased from PromoCell as non-activated, fully polarized M1 (GM-CSF) or M2 (M-CSF) cells (#C-12914, #C-12915; respectively). The primary human M1 and M2 macrophages were cultured on human fibronectin-coated vessels and maintained in M1- or M2-Macrophage Generation Medium XF (PromoCell; #C-28055, #C-28056), respectively, for 7 days according to the instruction manual. Then, M1 and M2 macrophages were activated as above for 2 days with IFNγ (20 ng/ml) and LPS (100 ng/ml), or IL-4 (20 ng/ml) and IL-10 (20 ng/ml), respectively. All cell lines were tested for mycoplasma prior to experiments. All cell lines were authenticated by the vendor prior to purchase and tested for mycoplasma using the MycoAlert kit (#LT07, Lonza, Switzerland).

### RNA isolation, cDNA synthesis, and quantitative PCR

Total RNA was extracted from cells using RNeasy Plus Mini Kit (Qiagen; #74136) according to the manufacturer’s instructions. For quantitative PCR (qPCR), cDNA was synthesized from 1 µg total RNA with SuperScript IV VILO Master Mix (Invitrogen; #11766050).

PCRs were performed on cDNA samples using TaqMan Gene Expression assays and TaqMan Fast Advanced Master Mix (Applied Biosystems; #4444556) according to the manufacturer’s protocol. All TaqMan assays used in our experiments are listed in Supplementary Table 1. TaqMan qPCR was set up and run on the 7500 Fast Real-Time PCR System (Applied Biosystems; Foster City, CA, USA). Relative mRNA expression of target genes was analyzed after normalization to the reference gene *HPRT1* using the ∆∆Ct method (calculated as fold change compared to untreated THP-1 or CD14^+^ hPBM cell samples). All qPCR analyses are based on at least three experimental replicates, with three technical replicates for each.

### Western blot

Total cell extracts were prepared by resuspending the harvested cells in ice-cold RIPA lysis buffer (50 mM Tris–HCl, pH 8.0, 150 mM NaCl, 5 mM EDTA, 0.5% (v/v) Nonidet P-40, 0.5% (v/v) Triton X-100, 0.1% sodium deoxycholate, 2 mM Na_3_VO_4_, 1 mM DTT, and 1x EDTA-free Protease Inhibitor Cocktail (Roche; #11873580001)). The extracts were incubated for 10 min on ice, then centrifuged at 10,000×*g* for 10 min at 4 °C. Protein concentration was measured, and 30 µg was separated on a 4–20% gradient SDS–PAGE gel. The proteins were then transferred onto a PVDF membrane (Bio-Rad Laboratories; #1620175). After blocking with StartingBlock T20 buffer (Thermo Scientific; #37543), the membrane was probed with the primary antibodies (Supplementary Table 2). The Secondary antibodies were sheep anti-mouse and sheep anti-rabbit HRP-linked antibodies (Abcam; #ab6808, #ab6795; respectively). Signal detection was with ECL Western Blotting Substrate and light-sensitive films (Thermo Scientific; #32106 and #34089, respectively). Finally, semi-quantification of the bands was performed single-blinded using ImageJ 1.53 K software (NIH; Bethesda, MD, USA). Uncropped full-size Western blot images are shown in Supplementary fig. S1.

### Cytokine profiling

Cytokine profile analysis of cell culture media from THP-1-derived macrophages was performed using V-PLEX Human SAA Kit, V-PLEX Angiogenesis Panel 1 Human Kit (containing TIE-2, bFGF, PlGF, VEGF-A, VEGF-C, VEGF-D and VEGFR-1/Flt-1) and U-PLEX Biomarker Group 1 (hu) Assays (G-CSF, GM-CSF, TNF-α, TNF-β, IL-1β, IL-6, IL-10, IL-18, IL-23 and IL-27) (all from Meso Scale Discovery; #K151SSD-1, #K15190D-1, #K15067L-1; respectively) according to the manufacturer’s protocol. The samples were read on a MESO QuickPlex SQ 120 instrument, and the data were analyzed using Discovery Workbench 4.0 software (both from Mesoscale Discovery).

### Apoptosis and proliferation assays

To assess apoptosis in THP-1 monocytes and their derived macrophages, Annexin V-FITC Apoptosis Detection Kit (Sigma-Aldrich; #APOAF) was used according to the manufacturer’s recommendations. The proliferative potential of 4T1-Luc cells was assessed using direct cell counting (with trypan blue exclusion) 48 h after treatment with conditioned medium and evaluated as a percentage of the M1 control condition. Proliferation of THP-1 monocytes, MDA-MB-231, and MDA-MB-468 cells was determined with Click-iT EdU Alexa Fluor 647 Flow Cytometry Assay Kit (Invitrogen; #C10424) according to the experimental protocol of the manufacturer. Detection of the proliferating cells with active DNA synthesis in S-phase of the cell cycle was based on a click reaction with the fluorescent dye Alexa Fluor 647. The samples were analyzed by flow cytometry, and the proliferation index was calculated as the ratio of the percentage of the proliferating cells to that of the non-proliferating cells. CytoFLEX S flow cytometer (Beckman Coulter; Brea, CA, USA) and its accompanying software CytExpert (version 2.5) were used for both assays.

### Invasion assay

Matrigel invasion chambers (Corning; #354480) were hydrated with warm starving medium (RPMI-1640 supplemented with 0.1% FBS) and incubated for 2 h at 37 °C. Then, for each chamber, 1 × 10^5^ MDA-MB-231 cells were seeded in the Matrigel insert in 500 µl of the starving medium, while the bottom well was filled with 750 µl of conditioned RPMI-1640 medium (from in vitro cultured M1 or M2 macrophages differentiated from THP-1 monocytes and treated with/without DEX from day 6 to day 8 of the previously described protocol). Complete RPMI-1640 medium (containing 10% FBS) was used as a positive control, and the starving medium as a negative control. After 24 h, migrated cells at the lower surface of the Matrigel insert were fixed with 4% (v/v) paraformaldehyde for 10 min, stained with 0.33% (w/v) crystal violet for 30 min, imaged, and then counted with ImageJ 1.53K software. Percent invasion was calculated from the ratio between the mean number of invaded cells in the treated conditions to the mean number of invaded cells in the negative control condition.

### Animal experiments

Six-week-old female wild-type BALB/cJRj mice were purchased from Janvier Labs (Le Genest-Saint-Isle, France) and randomly split into experimental groups containing 9 animals each. After acclimatization for 10 days at the animal facility, 4 × 10^5^ 4T1-Luc2 cells were suspended in 100 µl DPBS (lacking Ca^2+^ and Mg^2+^) and injected into the 4th mammary fat pad. Control mice (3 mice) received only DPBS injection. Upon palpable tumor formation, on day 14 of the experiment, each test mouse was treated with one weekly intraperitoneal (i.p.) injection of 20 mg/kg paclitaxel (PXL, Sigma-Aldrich; #T7402) on 3 successive weeks, and/or subcutaneous (s.c.) implantation of a 21-day-release dexamethasone (DEX) pellet (0.125 mg/day, in total 131.25 mg over 21 days) or placebo (PBO) pellet (Innovative Research of America; #G-131 and #C-111; respectively). Vehicle-treated mice received weekly i.p. injections of a vehicle solution (VEH) composed of 10% Cremophor EL (Calbiochem, #238470), 10% ethanol, and 80% PBS.

To monitor the progression of the disease, the tumor volume was measured with a caliper throughout the experiment. At the endpoint (on day 42), the mice were imaged for bioluminescence from 4T1-Luc2 tumor cells on an IVIS SpectrumCT instrument (PerkinElmer; Waltham, MA, USA) after i.p. injection of D-luciferin (150 mg/kg) (Caliper; #122796). The Images were analyzed single-blinded, and the luminescent signals were measured as photons per second, with Living Image 4.5 software (PerkinElmer). Mice that reached endpoint due to tumor size above 2000 mm^3^ before day 42 were sacrificed. Tissues from days 37 and 42 were used for analyses.

For micro-CT imaging, lungs from test and control mice were stained with 1% iodine solution as a contrast dye, then scanned using a Quantum GX2 CT scanner (PerkinElmer). The scans were made under tube voltage of 90 kV, tube current of 200 μA, field of view at 20 mm, and scan time of 4 min. The 3D µCT data were reconstructed and analyzed single-blinded using Analyze 12.0 software (Biomedical Imaging Resource, Mayo Clinic; Rochester, MN, USA). The IVIS and µCT procedures and analyses were performed by research staff blinded to the treatments. A semi-automatic segmentation was used to define the total tumor volume and the total lung volume, based on X-ray attenuation difference between the tumor tissue and the lung parenchyma after iodine staining. Control mice, without tumor burdens, served as a baseline for the µCT analyses. The mice were anesthetized in most experimental procedures (cell injections, pellet implantations, and imaging applications). All in vivo procedures were approved by the local ethics committee (Linköpings Djurförsöksetiska Nämnd) under the ethical approval numbers 05999-2020 and 2202-2021. Mice that reached humane endpoint before the end of the experiment (e.g., maximum tumor size) were not included in the analysis.

### Immunostaining

Tissue samples from the mouse mammary tumors were fixed with 4% paraformaldehyde, embedded in paraffin, and then cut into 4 µm-thick sections. After deparaffinization and hydration, antigen-retrieval was with sodium citrate buffer (10 mM sodium citrate, pH 6.0) in a pressure steamer at 121 °C for 20 min, followed by blocking of non-specific staining using 10% goat serum (Thermo Fisher; #PCN5000) in PBS for 1 h at room temperature. After that, the sections were incubated overnight at 4 °C with the optimal dilutions of anti-COX-2 or anti-CD163 antibodies (Supplementary Table 2). The sections were then washed with PBS and stained with Alexa Fluor 488-conjugated goat anti-rabbit IgG (Invitrogen; #A-11008) secondary antibodies at a 1:1000 dilution for 1 h at room temperature. Finally, the slides were counterstained with DAPI nuclear dye (Thermo Fisher; #D1306) at 300 nM concentration for 10 min, then coverslipped. For immunohistochemistry, the lungs were processed as described earlier [33] using vimentin antibody (Leica; #NCL-VIM-V9) at 1:800 dilution, ABC-HRP and Impact-DAB kits (Vector Laboratories; #PK400, #SK4100; respectively). Imaging was on an Axioplan-2 microscope (Carl Zeiss; Göttingen, Germany), and the images were captured with Zeiss AxioVision 4.0 software. The image analysis was performed using ImageJ 1.53K software. For immunofluorescence, positive cells were enumerated on four randomly chosen microscopic fields at ×20 magnification.

### Bulk RNA sequencing analysis

Total RNA was extracted in our lab as described above. Library preparation and Illumina paired-end sequencing were performed by Novogene. First, RNA quality was analyzed by 1% gel electrophoresis, NanoPhotometer (Implen; CA, USA), and on Bioanalyzer 2100 (Agilent Technologies; CA, USA). For cDNA library preparation, 1 µg RNA per sample was used with NEBNext Ultra RNA Library Prep Kit for Illumina (New England Biolabs; #E7530) following the manufacturer’s recommendations, and index codes were added to attribute sequences to each sample. The PCR products were purified by an AMPure XP system (Beckman Coulter), and the library quality was assessed on an Agilent Bioanalyzer 2100. The clustering of the index-coded samples was performed on a cBot Cluster Generation System using PE Cluster Kit cBot-HS (Illumina) according to the manufacturer’s instructions. After cluster generation, the library preparations were sequenced on an Illumina HiSeq 2000 platform, and paired-end reads were generated.

Clean reads of FASTQ format were obtained by removing reads containing adapter and poly-N sequences and reads with low quality from the raw data. At the same time, Q20, Q30, and GC content of the clean data were calculated. Paired-end clean reads were then mapped to GRCh38/hg19 human reference genome using HISAT2 software (v2.0.5). The Cufflinks Reference Annotation Based Transcript (RABT) assembly method was used to assemble the set of transcript isoforms of each bam file obtained in the mapping step. Read numbers were counted using HTSeq, followed by FPKM (the expected numbers of Fragments Per Kilobase of transcript sequence per Million base pairs sequenced) calculation. Differential expression analysis was performed using the DESeq2 R package. The resulting *P* values were adjusted using Benjamini and Hochberg’s approach for controlling the False Discovery Rate (FDR). Genes with an adjusted *P* value < 0.05 found by DESeq2 were assigned as differentially expressed. Shared gene functions were identified with DAVID (https://david.ncifcrf.gov) [34] annotation and visualization database using gene ontology (GO) enrichment and Kyoto encyclopedia of genes and genomes (KEGG) analyses of differentially expressed genes. GO and KEGG terms with corrected *P* value < 0.05 were considered significantly enriched. Visualization of data was performed with R (DESeq package), Heatmapper (http://www.heatmapper.ca/) [35], VolcaNoseR (https://huygens.science.uva.nl/VolcaNoseR2/) [36], and the Venn diagram web tool at the University of Gent (https://bioinformatics.psb.ugent.be/webtools/Venn/).

### Statistical analysis

All statistical analyses were performed using Prism 9.02 (GraphPad Software; Boston, MA, USA) and were based on at least 3 biological or experimental replicates. The required number of mice was calculated by performing power analysis based on a pilot experiment. Data were tested for equal variance by *F*-test. An unpaired two-tailed Student’s *t*-test was used to compare the two groups. Unless stated otherwise, multiple group analyses were performed by one-way or two-way analysis of variance (ANOVA), followed by post-hoc test for multiple comparisons as indicated in the figure legends. The results are expressed as mean ± SD or ±SEM. *P* < 0.05 (*) was considered statistically significant.

### Ethics

All animal experimentation was approved by the local ethics committee (Linköpings Djurförsöksetiska Nämnd) under the ethical approval numbers 05999-2020 and 2202-2021, and all methods were in accordance with relevant guidelines and regulations.

## Supplementary information

Supplemental material

Supplementary Table S3

Supplementary Table S4

Supplementary Table S5

## Data Availability

The data presented in this study are provided in the main article and in the supplementary materials. The RNA-seq data have been deposited in NCBI’s Gene Expression Omnibus under the accession number: GSE290519.

## References

[1] Shih A, Jackson KC 2nd. Role of corticosteroids in palliative care. J Pain Palliat Care Pharmacother. 2007;21:69–76. 18032321

[2] Cook AM, McDonnell AM, Lake RA, Nowak AK. Dexamethasone co-medication in cancer patients undergoing chemotherapy causes substantial immunomodulatory effects with implications for chemo-immunotherapy strategies. Oncoimmunology. 2016;5:e1066062. 27141331 10.1080/2162402X.2015.1066062 PMC4839331

[3] Call TR, Pace NL, Thorup DB, Maxfield D, Chortkoff B, Christensen J, et al. Factors associated with improved survival after resection of pancreatic adenocarcinoma: a multivariable model. Anesthesiology. 2015;122:317–24. 25305092 10.1097/ALN.0000000000000489

[4] Sun N, Ji H, Wang W, Zhu Q, Cao M, Zang Q. Inhibitory effect of dexamethasone on residual Lewis lung cancer cells in mice following palliative surgery. Oncol Lett. 2016;13:356–62. 28123567 10.3892/ol.2016.5422 PMC5244974

[5] Zhang D, Zhang Y, Cai Z, Tu Y, Hu Z. Dexamethasone and lenvatinib inhibit migration and invasion of non‑small cell lung cancer by regulating EKR/AKT and VEGF signal pathways. Exp Ther Med. 2020;19:762–70. 31853327 10.3892/etm.2019.8225 PMC6909740

[6] Zhang Y, Shi G, Zhang H, Xiong Q, Cheng F, Wang H, et al. Dexamethasone enhances the lung metastasis of breast cancer via a PI3K–SGK1–CTGF pathway. Oncogene. 2021;40:5367–78. 34272474 10.1038/s41388-021-01944-w PMC8413128

[7] Obradovic MMS, Hamelin B, Manevski N, Couto JP, Sethi A, Coissieux MM, et al. Glucocorticoids promote breast cancer metastasis. Nature. 2019;567:540–4. 30867597 10.1038/s41586-019-1019-4

[8] Pang JM, Huang Y-C, Sun S-P, Pan Y-R, Shen C-Y, Kao M-C, et al. Effects of synthetic glucocorticoids on breast cancer progression. Steroids. 2020;164:108738. 33065150 10.1016/j.steroids.2020.108738

[9] Skytthe MK, Graversen JH, Moestrup SK. Targeting of CD163+ macrophages in inflammatory and malignant diseases. Int J Mol Sci. 2020;21:5497. 32752088 10.3390/ijms21155497 PMC7432735

[10] Yoshikawa K, Mitsunaga S, Kinoshita T, Konishi M, Takahashi S, Gotohda N, et al. Impact of tumor-associated macrophages on invasive ductal carcinoma of the pancreas head. Cancer Sci. 2012;103:2012–20. 22931216 10.1111/j.1349-7006.2012.02411.x PMC7659389

[11] Sica A, Schioppa T, Mantovani A, Allavena P. Tumour-associated macrophages are a distinct M2 polarised population promoting tumour progression: potential targets of anti-cancer therapy. Eur J Cancer. 2006;42:717–27. 16520032 10.1016/j.ejca.2006.01.003

[12] Hunt H, Donaldson K, Strem M, Zann V, Leung P, Sweet S, et al. Assessment of safety, tolerability, pharmacokinetics, and pharmacological effect of orally administered CORT125134: an adaptive, double-blind, randomized, placebo-controlled phase 1 clinical study. Clin Pharmacol Drug Dev. 2018;7:408–21. 28967708 10.1002/cpdd.389 PMC5947602

[13] Jumeau C, Awad F, Assrawi E, Cobret L, Duquesnoy P, Giurgea I, et al. Expression of SAA1, SAA2 and SAA4 genes in human primary monocytes and monocyte-derived macrophages. PLoS ONE. 2019;14:e0217005. 31100086 10.1371/journal.pone.0217005 PMC6524798

[14] Cassetta L, Fragkogianni S, Sims AH, Swierczak A, Forrester LM, Zhang H, et al. Human tumor-associated macrophage and monocyte transcriptional landscapes reveal cancer-specific reprogramming, biomarkers, and therapeutic targets. Cancer Cell. 2019;35:588–602.e10. 30930117 10.1016/j.ccell.2019.02.009 PMC6472943

[15] Murakami M, Zheng Y, Hirashima M, Suda T, Morita Y, Ooehara J, et al. VEGFR1 tyrosine kinase signaling promotes lymphangiogenesis as well as angiogenesis indirectly via macrophage recruitment. Arterioscler Thromb Vasc Biol. 2008;28:658–64. 18174461 10.1161/ATVBAHA.107.150433

[16] Wu L, Yan J, Bai Y, Chen F, Zou X, Xu J, et al. An invasive zone in human liver cancer identified by Stereo-seq promotes hepatocyte–tumor cell crosstalk, local immunosuppression and tumor progression. Cell Res. 2023;33:585–603. 37337030 10.1038/s41422-023-00831-1 PMC10397313

[17] Pan D, Kocherginsky M, Conzen SD. Activation of the glucocorticoid receptor is associated with poor prognosis in estrogen receptor-negative breast cancer. Cancer Res. 2011;71:6360–70. 21868756 10.1158/0008-5472.CAN-11-0362 PMC3514452

[18] Pang D, Kocherginsky M, Krausz T, Kim SY, Conzen SD. Dexamethasone decreases xenograft response to Paclitaxel through inhibition of tumor cell apoptosis. Cancer Biol Ther. 2006;5:933–40. 16775428 10.4161/cbt.5.8.2875

[19] Ruffell B, Affara NI, Coussens LM. Differential macrophage programming in the tumor microenvironment. Trends Immunol. 2012;33:119–26. 22277903 10.1016/j.it.2011.12.001 PMC3294003

[20] Wyckoff J, Wang W, Lin EY, Wang Y, Pixley FJ, Stanley ER, et al. A paracrine loop between tumor cells and macrophages is required for tumor cell migration in mammary tumors. Cancer Research. 2004;64:7022–9. 15466195 10.1158/0008-5472.CAN-04-1449

[21] Zheng P, Luo Q, Wang W, Li J, Wang T, Wang P, et al. Tumor-associated macrophages-derived exosomes promote the migration of gastric cancer cells by transfer of functional apolipoprotein E. Cell Death Disease. 2018;9. 10.1038/s41419-018-0465-5 PMC5864742 29567987

[22] Chanmee T, Ontong P, Konno K, Itano N. Tumor-associated macrophages as major players in the tumor microenvironment. Cancers. 2014;6:1670–90. 25125485 10.3390/cancers6031670 PMC4190561

[23] Yang L, Han P, Cui T, Miao Y, Zhao T, Cui Z, et al. M2 Macrophage inhibits the antitumor effects of lenvatinib on intrahepatic cholangiocarcinoma. Front Immunol. 2023;14. 10.3389/fimmu.2023.1251648 PMC10556255 37809069

[24] Escoter-Torres L, Caratti G, Mechtidou A, Tuckermann J, Uhlenhaut NH, Vettorazzi S. Fighting the fire: mechanisms of inflammatory gene regulation by the glucocorticoid receptor. Front Immunol. 2019;10:1859. 31440248 10.3389/fimmu.2019.01859 PMC6693390

[25] Brady ME, Sartiano GP, Rosenblum SL, Zaglama NE, Bauguess CT. The pharmacokinetics of single high doses of dexamethasone in cancer patients. Eur J Clin Pharmacol. 1987;32:593–6. 3653229 10.1007/BF02455994

[26] Rutz HP. Effects of corticosteroid use on treatment of solid tumours. The Lancet. 2002;360:1969–70. 10.1016/S0140-6736(02)11922-2 12493280

[27] Luvanda MK, Posch W, Vosper J, Zaderer V, Noureen A, Lass-Flörl C, et al. Dexamethasone promotes *Aspergillus fumigatus* growth in macrophages by triggering M2 repolarization via targeting PKM2. J Fungi. 2021;7:70. 10.3390/jof7020070 PMC7909285 33498318

[28] Smith HL, Foxall RB, Duriez PJ, Teal EL, Hoppe AD, Kanczler JM, et al. Comparison of human macrophages derived from peripheral blood and bone marrow. J Immunol. 2025;214:714–25. 40073092 10.1093/jimmun/vkae032 PMC12041772

[29] Xu L, Xia H, Ni D, Hu Y, Liu J, Qin Y, et al. High-dose dexamethasone manipulates the tumor microenvironment and internal metabolic pathways in anti-tumor progression. Int J Mol Sci. 2020;21. 10.3390/ijms21051846 PMC7084511 32156004

[30] Khosravi M, MoriBazofti H, Mohammadian B, Rashno M. The effects of the differentiated macrophages by dexamethasone on the immune responses. Int Immunopharmacol. 2023;124:110826. 37607463 10.1016/j.intimp.2023.110826

[31] Thorn CF, Whitehead AS. Differential glucocorticoid enhancement of the cytokine-driven transcriptional activation of the human acute phase serum amyloid a genes, *SAA1* and *SAA2*. J Immunol. 2002;169:399–406. 12077270 10.4049/jimmunol.169.1.399

[32] Gaiser AK, Bauer S, Ruez S, Holzmann K, Fändrich M, Syrovets T, et al. Serum amyloid A1 induces classically activated macrophages: a role for enhanced fibril formation. Front Immunol. 2021;12:691155. 34276683 10.3389/fimmu.2021.691155 PMC8278318

[33] Töhönen V, Antonson P, Boggavarapu NR, Ali H, Motaholi LA, Gustafsson J, et al. Transcriptomic profiling of the oocyte–cumulus–granulosa cell complex from estrogen receptor β knockout mice. F S Sci. 2024;5:306–17. 39168303 10.1016/j.xfss.2024.08.004

[34] Sherman BT, Hao M, Qiu J, Jiao X, Baseler MW, Lane HC, et al. DAVID: a web server for functional enrichment analysis and functional annotation of gene lists (2021 update). Nucleic Acids Res. 2022;50:W216–w21. 35325185 10.1093/nar/gkac194 PMC9252805

[35] Babicki S, Arndt D, Marcu A, Liang Y, Grant JR, Maciejewski A, et al. Heatmapper: web-enabled heat mapping for all. Nucleic Acids Res. 2016;44:W147–53. 27190236 10.1093/nar/gkw419 PMC4987948

[36] Goedhart J, Luijsterburg MS. VolcaNoseR is a web app for creating, exploring, labeling and sharing volcano plots. Sci Rep. 2020;10:20560. 33239692 10.1038/s41598-020-76603-3 PMC7689420

