# Supplementary material for: Dexamethasone drives macrophage repolarization linked to increased triple-negative breast cancer aggressiveness
Source: Cell Death Dis. 2025 Dec 19;17(1):114. doi: 10.1038/s41419-025-08363-9 (PMC12847883; doi:10.1038/s41419-025-08363-9)
Supplement: Supplementary file 1 — Supplemental material [file 41419_2025_8363_MOESM1_ESM.pdf]

SUPPLEMENTARY MATERIAL

Supplementary Figure S1. Uncropped full-size of Western blot membranes.

Supplementary Figure S2. Glucocorticoid receptor (GR)/*NR3C1* and the pan-macrophage marker *CD14* expression in M1 and M2 macrophages.

Supplementary Figure S3. Apoptosis and proliferation analyses.

Supplementary Figure S4. Hierarchical clustering.

Supplementary Table S1. TaqMan Gene Expression assays (Applied Biosystems).

Supplementary Table S2. List of primary antibodies.

Supplementary Table S3. DEGs, FPKM across conditions.

Supplementary Table S4. Fold change DEGs

Supplementary Table S5. Pathway analysis

Supplementary Figure S1

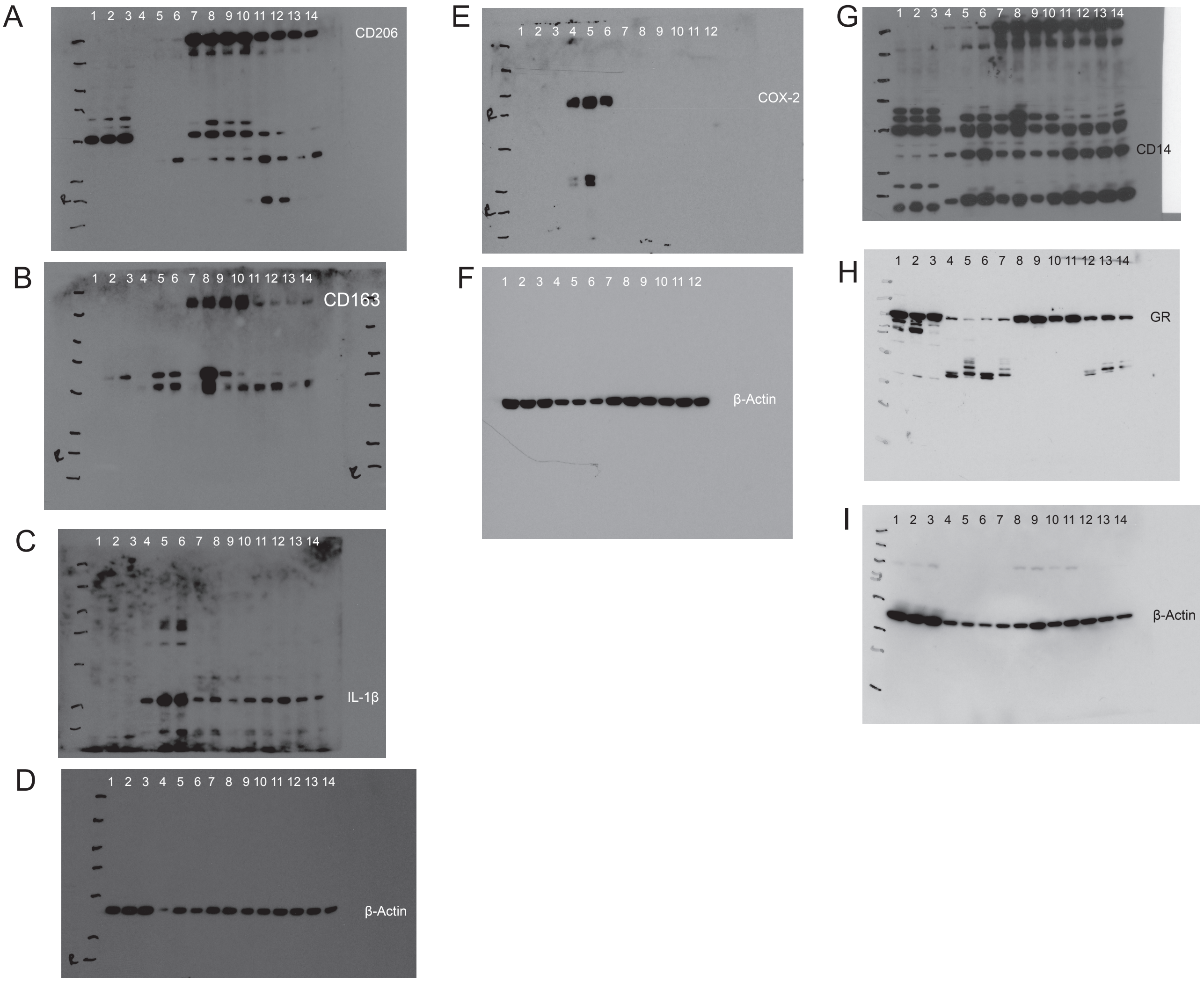

Supplementary Figure S1. Uncropped full-size of Western blot membranes.
A-D, Western blot analysis of CD206, CD163, IL-1β, and β-Actin. Lanes 1-3,THP-1 cells; 4-6, M1 cells; 7-10, M1 cells treated with 100 μM DEX; and 11 - 14, and M2 polarized macrophages.
E-F, Western blot analysis of COX-2, and β-Actin. Lanes 1-3,THP-1 cells; 4-6, M1 cells; 7-9, M1 cells treated with 100 μM DEX; and 10 - 12, and M2 polarized macrophages.
G-I, Western blot analysis of CD14, GR and β-Actin. Lanes 1-3,THP-1 cells; 4-7, M1 cells; 8-11, M1 cells treated with 100 μM DEX; and 12 - 14, and M2 polarized macrophages.

Supplementary Figure S2

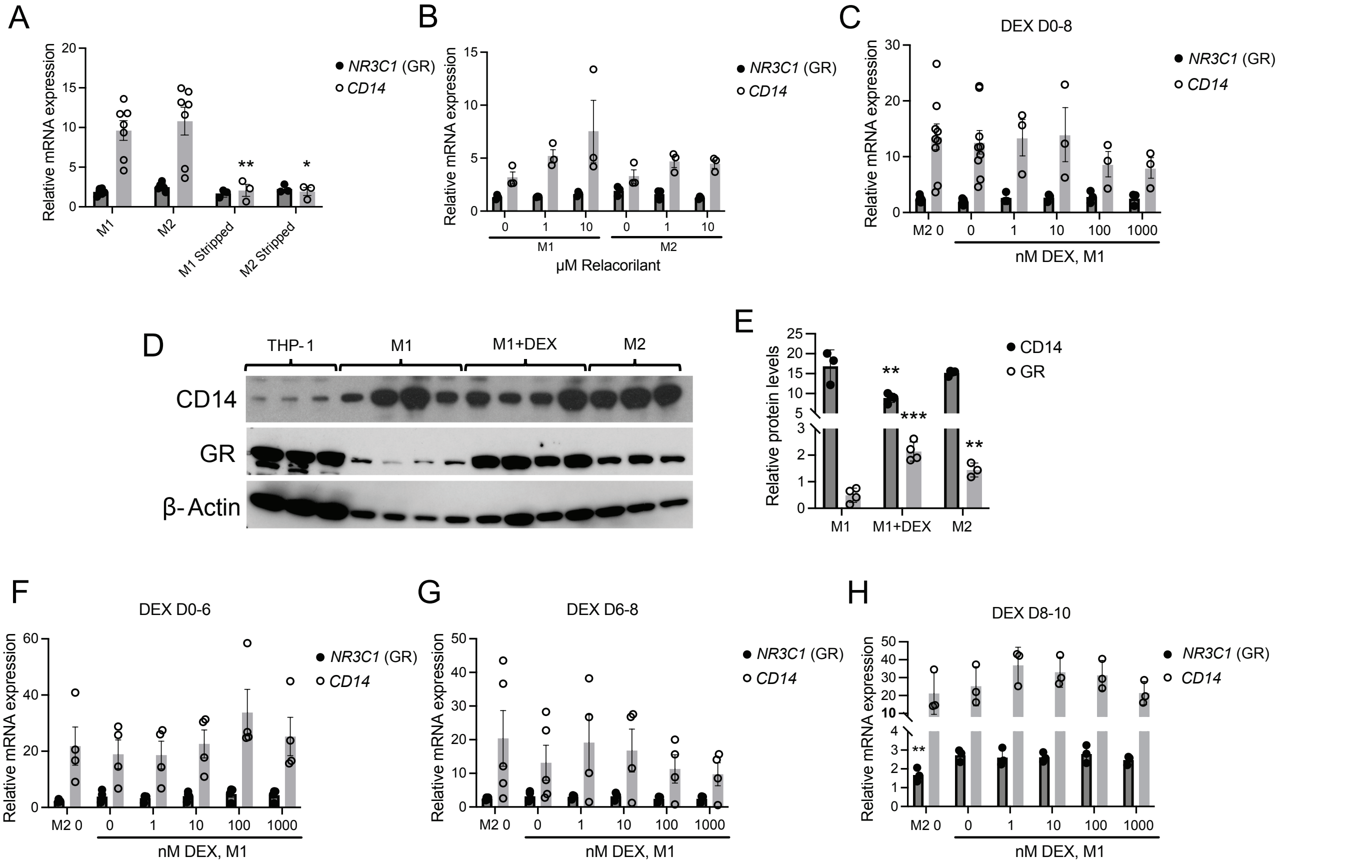

Supplementary Figure S2. Glucocorticoid receptor (GR)/*NR3C1* and the pan-macrophage marker *CD14* expression in M1 and M2 macrophages. A) Relative mRNA expression of *NR3C1* (GR gene) and the pan-macrophage marker *CD14* (n=3 [stripped], 6 [normal]) upon culturing in normal or steroid-stripped medium during 8 days of differentiation. B) Relative mRNA expression of *NR3C1*/GR and CD14 (n=3) upon increasing concentrations of relacorilant in M1 or M2 differentiating conditions, and C) upon M1 differentiation in the presence of increasing concentrations of dexamethasone (DEX), or M2 differentiation in the absence of DEX (n=3, 9 [M2 0, M1 0]). D) Western blots of the pan-macrophage marker CD14 and GR in THP-1 cells, M1 and M2 polarized macrophages treated with vehicle (EtOH) or 100 μM DEX (DEX). E) Western blot analysis of CD14 and GR relative to THP-1 cells (n=3, 4). Comparisons were to M1 EtOH. F) Relative mRNA expression of *NR3C1*/GR and *CD14* upon M1 differentiation in the presence of increasing concentrations of DEX during F) day 0-6 of M1 differentiation (n=4), G) day 6-8 of M1 differentiation (n=4, 5 [M2 0, M1 0]), or H) day 8-10 of M1 differentiation (n=3). All comparisons were to M1 polarized macrophages at 0 nM DEX, and all mRNA expression was relative to untreated THP-1 cells and normalized to the housekeeping gene *HPRT1*. Bars represent mean ± SEM in A-C and F-H, or ±SD in E, * $P < 0.05$, ** $P < 0.01$, *** $P < 0.001$. Statistical significance was determined using one-way ANOVA followed by Dunnett's multiple comparisons correction.

Supplementary Figure S3

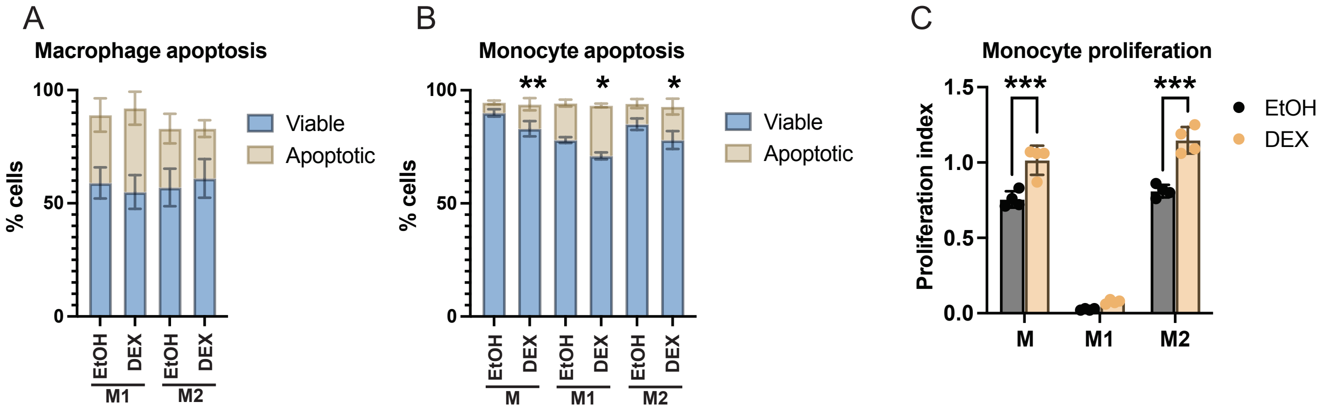

Supplementary Fig. S3. Apoptosis and proliferation analyses. Percent apoptotic and viable A) macrophages and B) monocytes, as well as C) monocyte proliferation upon vehicle (EtOH) or 100 μM DEX treatment under M, M1, and M2 differentiating conditions. Comparisons were between vehicle and DEX treatments, and bars represent mean ± SD, * $P < 0.05$, ** $P < 0.01$, *** $P < 0.001$. Statistical significance was determined using two-way ANOVA followed by Tukey's (in A and B) or Šídák's multiple comparisons correction (in C).

Supplementary Figure S4

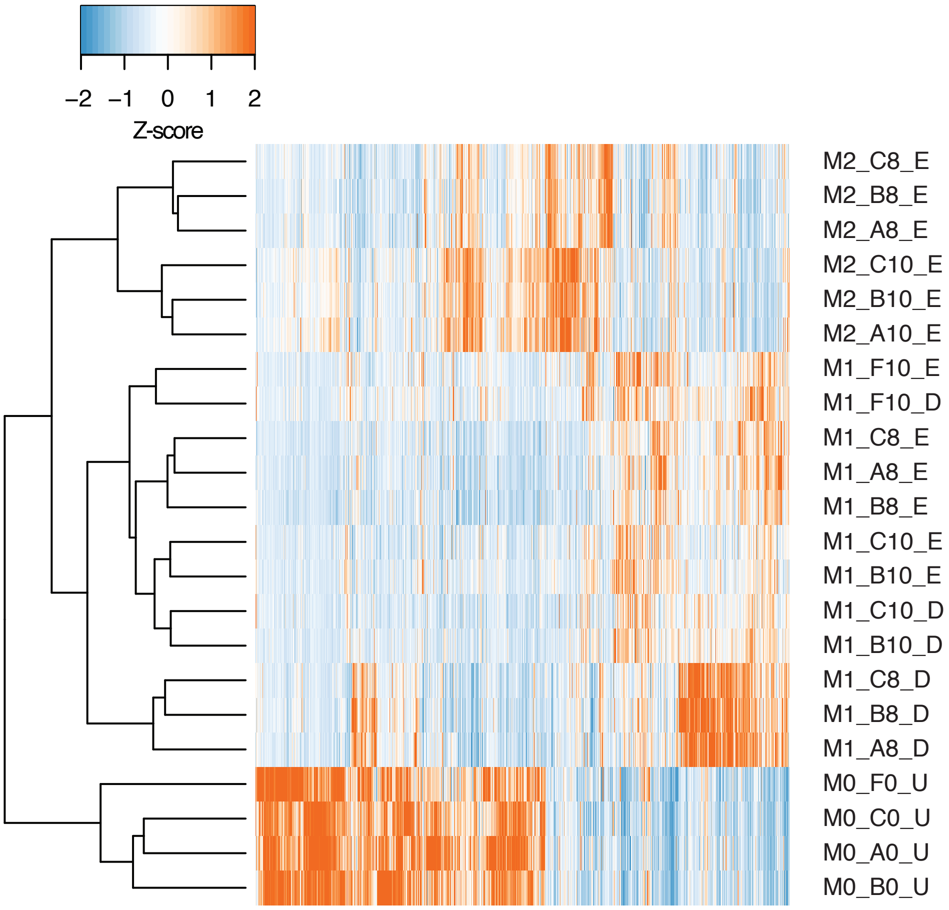

Supplementary Figure S4. Hierarchical clustering of all DEGs between THP-1 monocytes (M0) and macrophages (M1 and M2), treatment groups (E, EtOH, vehicle; D, DEX; U, untreated; 8=from D0 to D8; 10=from D8 to D10; A, B, C and F refers to experimental replicates). Expression values are Z-score-transformed and clustered using complete linkage and Euclidean distance.

Supplementary Table 1. TaqMan Gene Expression assays (Applied Biosystems).

Target gene	Other names	Species	TaqMan assay ID	Catolog #
HPRT1		Human	ARPRKZ6	4351372
NR3C1	GR	Human	Hs00353740_m1	4331182
CD14		Human	Hs00169122_g1	4331182
CCR7		Human	ARMFX4W	4351372
CD80	B7-1, CD28LG1	Human	Hs01045161_m1	4331182
PTGS2	COX-2	Human	Hs00153133_m1	4331182
IL1B	IL-1β	Human	Hs01555410_m1	4331182
CD163		Human	Hs00174705_m1	4331182
MRC1	CD206, MMR, hMR	Human	Hs00267207_m1	4331182

Supplementary Table 2. List of primary antibodies.

Target	Clone	Vender	Catolog #	Class	Dilution	Application
GR		Abcam	ab3579	Polyclonal	1:400	WB
CD14	EPR3653	Abcam	ab133335	Monoclonal	1:5000	WB
COX-2	EPR8588	Abcam	ab151571	Monoclonal	1:1000	WB
COX-2	EPR12012	Abcam	ab179800	Monoclonal	1:100	IF
IL-1β		Abcam	ab9722	Polyclonal	0.2 µg/ml	WB
MR/CD206		Abcam	ab64693	Polyclonal	1:1000	WB
CD163	EPR19518	Abcam	ab182422	Monoclonal	1:1000<br>1:100	WB<br>IF
Actin	C4	Merk	MAB1501	Monoclonal	1:10000	WB
Vimentin	V9	Leica	NCL-VIM-V9	Monoclonal	1:800	IHC
